# Heart Rate Expenditure Correlates with Right Ventricular Function

**DOI:** 10.1513/AnnalsATS.201909-683RL

**Published:** 2020-03

**Authors:** Daniel J. Lachant, Allison N. Light, Maria L. Mackin, Ronald G. Schwartz, R. James White

**Affiliations:** ^1^ University of Rochester Medical Center Rochester, New York

*To the Editor*:

Right ventricular (RV) function is the strongest predictor of morbidity (1) and mortality (2) in pulmonary arterial hypertension (PAH), and other measures of prognosis and therapy response are related to RV function (3–5). The 6-minute-walk test (6MWT) is a core component in the evaluation of PAH and probably indirectly reflects RV function during stress. Continuous heart rate measures during 6MWT could increase the utility of the test by focusing on the cardiac expenditure during exercise.

The objective of this study was to determine whether *1*) cadmium-zinc-telluride (CZT) single-photon emission computed tomography (SPECT) equilibrium radionuclide angiocardiography (ERNA) would detect improved RV function after intensified therapy for PAH and *2*) continuous heart rate monitoring during 6MWT would improve the utility of the 6MWT.

## Methods

### Study population

All study procedures were approved by the University of Rochester research subjects review board, and each participant provided written consent. As patients presented to our Pulmonary Hypertension Association–accredited comprehensive care center, we enrolled treatment-naive participants with World Health Organization group 1 PAH or those with clinical indications for a third PAH therapy (12 total) in the “treatment intensification” group, and we enrolled 12 participants with PAH with varying degrees of RV failure in an age- and sex-matched stable group (no therapy change planned) (36 total screened; 24 enrolled). N-terminal prohormone brain natriuretic peptide (NT-proBNP) measurement, World Health Organization functional class assessment, 6MWT with continuous heart rate measurements, and cardiac imaging were all performed on the same day. In the treatment intensification group, testing was performed before adding therapy and again 6 months later. In the participants with stable PAH, testing occurred within a 2-week window to assess for reproducibility.

### Nuclear image acquisition and analysis

**Figure 1.**
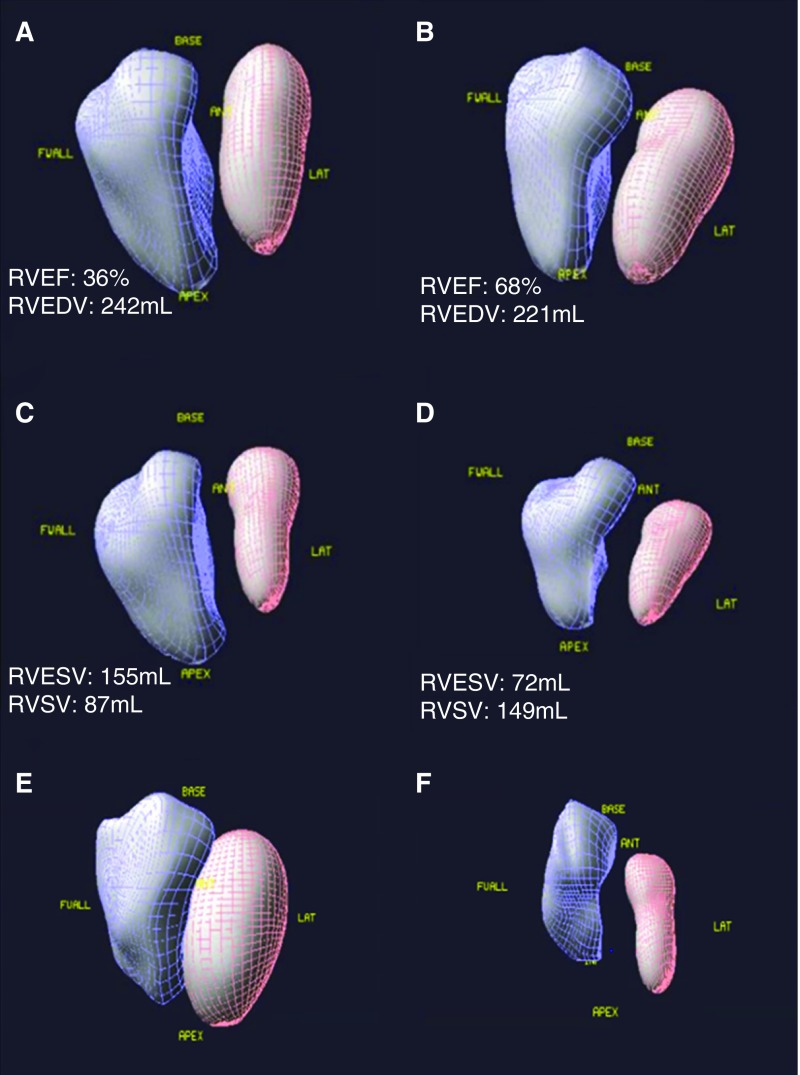
Ventricular images obtained by cadmium-zinc-telluride (CZT) single-photon emission computed tomography (SPECT) equilibrium radionuclide angiocardiography (ERNA). (*A*) The left (red) and right (blue) ventricles at end diastole in a treatment-naive participant. (*B*) The left and right ventricles at end diastole after 6 months of combination therapy with ambrisentan and tadalafil. (*C*) The left and right ventricles at end systole in the same treatment-naive participant. (*D*) The left and right ventricles at the end of systole after 6 months of combination therapy with ambrisentan and tadalafil. (*E*) CZT SPECT ERNA images excluded because of separation issues; the ventricles could not be resolved. (*F*) CZT SPECT ERNA images excluded because of low intensity (unable to be processed). RVEDV = right ventricular end-diastolic volume; RVEF = right ventricular ejection fraction; RVESV = right ventricular end-systolic volume; RVSV = right ventricular stroke volume.

We imaged the RV volumes using the D-SPECT CZT cardiac gamma camera (Spectrum Dynamics) (Figure 1), and all images were acquired using the same camera. Nonlabeled stannous pyrophosphate (2.4 mg) was injected intravenously 30 minutes before injection of Tc-99m-pertechnetate (10 mCi, ∼2.5 mSv). The images were analyzed using the Quantitative Blood Pool SPECT fully automated software (version 3.2.1; Cedars-Sinai Medical Center) (6). The algorithm for volume measurement is based on endocardial border determination (7). Manually directed automated reprocessing was required in 21 of 38 acceptable scans and was done as described in the Cedars-Sinai software manual and the video tutorial (www.csaim.com/tutorials.php#qbsmanual). All of the images were analyzed blindly and in random sequence by R.J.W., A.N.L., and R.G.S.

### Heart rate analysis

**Figure 2.**
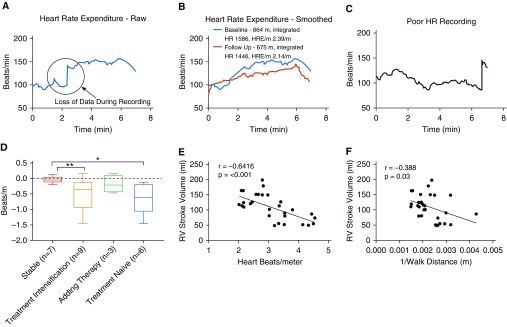
Heart rate (HR) monitoring during 6-minute-walk test (6MWT). (*A*) Baseline (blue) HR tracing during the 6MWT in a treatment-naive participant. Loss of recording occurred during the 6MWT (black circle). (*B*) Interpolating data where the HR signal was lost allowed heart rate expenditure (HRE) and HRE/d to be calculated. There was a significant decrease after adding ambrisentan and tadalafil. (*C*) An HR tracing that seemed physiologically improbable and was excluded from analysis. During the walk, HR was not accurately measured. (*D*) HRE/d decreased significantly in the treatment intensification group compared with the stable control group (**P < 0.02). This was primarily driven by the treatment-naive group (*P < 0.02). Despite the small sample size, improvement associated with adding a third therapy is apparent. Error bars calculated according to the Tukey’s method. (*E*) Right ventricular (RV) stroke volume correlated strongly with HRE/d (*r* = −0.64; *P* < 0.001). (*F*) RV stroke volume versus 1 divided by walk distance in 6MWT correlation was poor compared with RV stroke volume versus HRE/d. HRE/d = heart rate expenditure/distance walked in 6MWT.

The Alpha 2 athletic wristwatch (MiO) athletic wristwatch was used to measure continuous pulse during 6MWT. The AlphaMio2 measures pulse via photoplethysmography (Figure 2). To minimize data loss, one author blinded to the participant and sequence reviewed the pulse tracings to smooth tracings when recordings were clearly not physiologic (Figure 2). We excluded tracings that were excessively noisy or physiologically improbable (Figure 2). Heart rate expenditure (HRE) was calculated by integrating pulse during walk.

### Statistical analysis

Owing to small sample sizes, we compared data from the two groups using nonparametric testing: Wilcoxon rank-sum test for paired comparisons and Kruskal-Wallis test for three-way analysis of variance using Prism 7.0 software (GraphPad Software Inc.).

## Results

Twenty-four participants completed all testing with 19 of 24 used for paired analysis, 10 in the treatment intensification group and 9 in the stable control group (Table 1). Of the 19 participants with paired imaging, 11 of 38 scans required minor manual reprocessing on at least one scan to improve tracking of the right ventricle.

**Table 1.** Baseline demographics of participants with two equilibrium radionuclide angiocardiographic scans

	Control (*n* = *9*)	Treatment Intensification (*n* = *10*)
Age, yr	48 (29–77)	48 (29–77)
Sex		
Female	5 (56%)	6 (60%)
PAH		
Idiopathic	4 (44%)	4 (40%)
Associated	4 (44%)	5 (50%)
Familial	1 (11%)	1 (10%)
Baseline therapies		
None	0	6 (60%)
Amb + Tad	3 (33%)	3 (30%)
(Amb + Tad) + treprostinil		
Oral	0	1 (10%)
Inhaled	1 (11%)	0
Subcutaneous	5 (56%)	0
Therapies added		
None	9 (100%)	0
Amb	0	1 (10%)
Amb + Tad	0	6 (60%)
Treprostinil		
Oral	0	2 (20%)
Subcutaneous	0	2 (20%)
Functional class		
Baseline		
II/III	8/1	10/0
Follow-up		
II/III	8/1	10/0
6MWT		
Baseline, m	398 (305–659)	459 (233–664)
Follow-up, m	418 (278–638)	461 (314–675)
NT-proBNP		
Baseline	202 (50–1,130)	470 (50–4,367)
Follow-up	173 (50–2,472)	302 (50–1,298)

*Definition of abbreviations*: 6MWT = 6-minute-walk test; Amb = ambrisentan; Tad = tadalafil; NT-proBNP = N-terminal prohormone brain natriuretic peptide; PAH = pulmonary arterial hypertension.

Age is median (range); the remainder of the numbers in parentheses until 6MWT are percentages. For the 6MWT and NT-proBNP, these are medians (range). NT-proBNP is pg/ml.

Right ventricular ejection fraction (RVEF) increased in the treatment intensification group from 47% at baseline to 57% at 6 months (*P* = 0.003). RV stroke volume increased from 84 ml to 110 ml (*P* = 0.04). By comparison, in the control group, RVEF and RV stroke volume remained unchanged (56% vs. 57%; 114 ml vs. 118 ml). In the one participant with clinical worsening excluded from summary analysis, the RVEF dropped from 59% to 47%.

Sixteen participants had paired ERNA and continuous heart rate measurements, comprising nine in the treatment intensification group and seven in the stable control group. Total HRE adjusted for walk distance (HRE/d) decreased in the treatment intensification group (3.5 vs. 3.2 beats/m; *P* = 0.02), whereas it was unchanged in the control group (2.4 vs. 2.4 beats/m; *P* = 0.57). This parameter correlated better with resting RV stroke volume (*r* = −0.64; *P* <0.001) than with 1 divided by walk distance in 6MWT (*r* = −0.38; *P* = 0.03) or NT-proBNP (*r* = −0.33; *P* = 0.07) (Figure 2).

## Discussion

This prospective pilot study demonstrated that CZT SPECT ERNA and continuous pulse monitoring during 6MWT could detect improvement in RV function after initiating or intensifying PAH therapy and showed stability and reproducibility in our participants not changing therapy. SPECT ERNA offers volumetric and functional assessment of the right ventricle (8), with current CZT SPECT ERNA offering improved image quality with ultralow radiation exposure at 10 mCi (2.5 mSv; roughly equivalent to routine ventilation–perfusion lung scan [9]). Owing to poor tracking on one scan, three participants were excluded from paired analysis, and another participant was excluded because of movement artifact. Of the 48 scans, we thus had irrecoverable errors on 4; especially given the prospective nature of this study, this is not unreasonable data loss. We hope to further refine the methodology to achieve no loss in future acquisitions. The user input for analysis is much less than for cardiac magnetic resonance imaging (no manual tracing at all) and therefore provides less opportunity for bias.

Using continuous pulse measurement at the wrist, we also observed dynamic changes in heart rate during 6MWT and treatment-related improvements with little or no change in 6MWT distance. We hypothesize that the decrease in HRE/d reflects improved RV function and is more sensitive than 6MWT distance alone for detecting improvement. HRE/d also correlated more tightly with resting RV function measured by CZT SPECT ERNA than two well-established noninvasive markers of RV function (NT-proBNP and 6MWT distance). Our preliminary data suggest that continuous measures of heart rate (especially HRE/d) could significantly increase the value of standardized 6MWT. These measurements (especially HRE/d) would logically attenuate the effects of mood or musculoskeletal pain that might heavily influence walk distance. Unfortunately, eight participants (33%) were excluded from analysis because of poor data quality obtained by photoplethysmography. Wrist-based measurements require adequate pulse strength and are sensitive to arm movement; we are exploring chest telemetry measurements to avoid these limitations.

Although prospective and rigorous in design, this is a small, single-center study, with substantial data loss (both CZT SPECT ERNA and photoplethysmography) limiting our interpretation. Nonetheless, we have shown two novel assessments that allowed us to detect changes after adding therapy compared with a stable group. Because RV function predicts long-term outcomes in PAH (10), developing accurate, reproducible, and readily available measures of RV function is critical to assess therapeutic response and improve outcomes.
